# Habitual fasting duration and accelerated multimorbidity in older adults

**DOI:** 10.1111/joim.70150

**Published:** 2026-08-20

**Authors:** Luis Kalmbach, David Abbad‐Gomez, Giorgi Beridze, Fernando Rodríguez‐Artalejo, Davide Liborio Vetrano, Amaia Calderón‐Larrañaga, Adrián Carballo‐Casla

**Affiliations:** ^1^ Aging Research Center Department of Neurobiology Care Sciences and Society Karolinska Institutet and Stockholm University Stockholm Sweden; ^2^ Department of Epidemiology and Evaluation Hospital del Mar Barcelona Spain; ^3^ Hospital del Mar Research Institute Barcelona Spain; ^4^ Research Network on Chronicity Primary Care and Health Promotion (RICAPPS) Instituto de Salud Carlos III (ISCIII) Madrid Spain; ^5^ PhD Program in Biomedicine Universitat Pompeu Fabra Barcelona Spain; ^6^ Department of Preventive Medicine and Public Health Universidad Autónoma de Madrid Madrid Spain; ^7^ Center for Networked Biomedical Research in Epidemiology and Public Health (CIBERESP) Madrid Spain; ^8^ IMDEA Institute of Nutrition Campus of International Excellence Madrid Spain; ^9^ Stockholm Gerontology Research Center Stockholm Sweden

**Keywords:** cohort studies, disease progression, feeding behavior, frail elderly, geriatrics, nutritional status

## Abstract

**Background:**

Prolonged fasting has been linked to metabolic benefits in younger adults, but its effects on chronic diseases in older populations remain unclear.

**Objective:**

To examine the association between habitual fasting duration and 15‐year chronic disease accumulation in older adults, overall, within organ systems, and between age groups.

**Methods:**

Data were drawn from the Swedish National study on Aging and Care in Kungsholmen, a longitudinal population‐based cohort of 2981 predominantly community‐dwelling adults aged ≥60 years. Habitual fasting duration was the longest interval between two typical eating occasions within a 24‐h window. Multimorbidity was the total number of chronic diseases (out of 60) at each visit, analyzed overall and within cardiovascular, neuropsychiatric, and musculoskeletal organ systems. Multivariate linear mixed models estimated the association between habitual fasting duration and the annual rate of multimorbidity accumulation, overall and stratified by age (<78 vs. ≥78 years).

**Results:**

Compared with the lowest habitual fasting quartile (6–11.5 h), participants in the highest quartile (14–24 h) experienced steeper annual accumulation of total chronic diseases (*β* = 0.119; 95% confidence interval: 0.070, 0.167), cardiovascular diseases (0.022 [0.006, 0.038]), and neuropsychiatric diseases (0.019 [0.005, 0.033]), but not musculoskeletal diseases. The association with total chronic disease accumulation was apparent in adults aged ≥78 years (*β* = 0.099 [0.031, 0.168]) but absent in those <78 years (−0.009 [−0.078, 0.061]). Findings remained consistent across sensitivity analyses addressing reverse causation, measurement bias, and residual confounding.

**Conclusion:**

In older adults, particularly the older old, longer habitual fasting duration was associated with faster chronic disease accumulation, suggesting it may be detrimental in advanced age.

## Graphical abstract

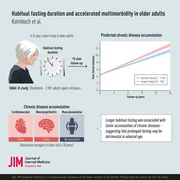

## Introduction

Age is a major risk factor for chronic diseases across organ systems [1]. With population aging accelerating worldwide [2], the number of people living with multiple chronic conditions (i.e., multimorbidity) is rising steadily, now affecting more than half of those aged 60 years and older [3, 4].

Multimorbidity is associated with functional decline, loss of autonomy, psychological distress, and an elevated mortality rate [5, 6, 7]. Among lifestyle factors, nutrition has long been recognized as a modifiable factor in chronic disease management [8], and recent longitudinal research highlights how dietary quality can influence the speed of chronic disease accumulation in older adults [9]. Emerging evidence also suggests that *when* people eat may affect long‐term health outcomes [10, 11], with prolonged fasting intervals probably linked to metabolic benefits [12, 13, 14].

Although fasting appears beneficial in younger populations [15], findings in older adults are limited and more nuanced. Zhang et al. [16] found that, in adults aged 60 and older, habitual fasting over 12.4 h was associated with increased cardiovascular mortality, whereas habitual fasting durations around 11.5 h were linked to the lowest overall mortality, suggesting that meal timing may be particularly sensitive in later life. A systematic review of intermittent fasting regimens in middle‐aged and older adults reported that short‐term trials of time‐restricted eating (14–16 h fasting per day) were associated with modest reductions in body weight, fat mass, and blood pressure, as well as improvements in glucose regulation [17]. However, observational studies in community‐dwelling older adults have shown that longer habitual fasting times are linked to adverse metabolic biomarkers and poorer lower extremity function [18, 19]. More broadly, a recent systematic review has emphasized that, although intermittent fasting can produce metabolic benefits in some contexts, robust evidence in humans is scarce and excessive fasting may entail health risks, underscoring the need for caution in older adults [20].

Even within older adults, differences may exist between the younger old and the older old, who may face additional nutritional and physiological challenges that could alter the health effects of prolonged fasting [21, 22, 23]. Yet, both subgroups have remained largely unexamined in previous research, raising the question of whether fasting‐related risks and benefits differ, not only between younger and older adults, but also within the older population itself.

Accordingly, the aim of this study was to examine whether habitual fasting duration is associated with the long‐term rate of multimorbidity accumulation in older adults. Using 15‐year longitudinal data from the Swedish National study on Aging and Care in Kungsholmen (SNAC‐K), we investigated these associations both overall and within homogeneous disease groups, namely, cardiovascular, neuropsychiatric, and musculoskeletal, while also exploring whether they differed between younger old (<78 years) and older old (≥78 years) adults.

## Methods

### Study design and participants

Our study is based on data from the SNAC‐K, an ongoing longitudinal cohort study conducted in Stockholm, Sweden [24]. The cohort includes randomly selected individuals aged 60 years and older, with baseline data collected between 2001 and 2004 (response rate: 73%). Participants aged <78 years are followed up every 6 years, whereas those aged ≥78 are assessed every 3 years to account for faster changes in health status with aging. The current analysis includes dietary data from Waves 1 to 3 (around 6 years) and multimorbidity data from Waves 1 to 6 (approximately 15 years).

Of the 3363 individuals participating at baseline, 382 had no mealtime information at Wave 1 and were excluded, resulting in a final analytical sample of 2981 participants. Information on follow‐up participation and dropout is summarized in Fig. S1.

The SNAC‐K study was approved by the Regional Ethical Review Board in Stockholm. Written informed consent was obtained from all participants or their next of kin at each study visit.

### Variables

#### Fasting operationalization

Throughout this study, fasting duration refers to the observed interval between eating occasions rather than a deliberate dietary practice. Habitual fasting duration was derived from self‐reported mealtime data in the SNAC‐K food frequency questionnaire, which asked for the time and content of up to eight typical daily eating occasions over the previous year. The questionnaire allowed reporting of main meals (breakfast, lunch, and dinner), as well as snacks (morning, afternoon, and evening) and two custom eating occasions. For each eating occasion, participants could report the approximate time and select one or more predefined food types: nothing at all/not applicable, drink only, drink with sandwich, porridge/yogurt/salad, fruit, cooked meal, or other. The food frequency questionnaire also included a previously validated section on portion sizes and food and beverage consumption [25], which was used to estimate energy and nutrient intakes.

Mealtimes were checked for internal consistency across waves, and missing values were imputed by carrying forward (primarily) or backward (alternatively) values from the nearest available wave whenever participants reported having meals but did not provide corresponding mealtimes. Maximum habitual fasting duration was calculated as the longest time gap between any two eating events within a 24‐h window, as derived from food frequency questionnaire‐reported mealtimes. As we lacked data on meal duration, it was assumed to be zero (e.g., if three meals were recorded at 8 a.m., 1 p.m., and 6 p.m., habitual fasting time was set to 14 h). Habitual fasting duration was modeled both as a continuous variable (per 1‐h increase) and categorical variable (four quartile‐like groups chosen to ensure group sizes as similar as possible): 6–11.5 h (reference; 23.5% of participants), 11.5–12.75 h (24.8% *of participants*), 12.75–14 h (29.7% *of participants*), and 14–24 h (22.0% *of participants*).

#### Multimorbidity

Diagnoses of chronic conditions in SNAC‐K are collected from multiple sources, including clinical assessments by physicians, self‐reported medical histories, laboratory measurements, and the use of certain medications (e.g., antidiabetics). At each wave, trained physicians conduct standardized assessments, which are supplemented by information from medical records and diagnostic data from the Swedish National Patient Register. In this study, we applied the disease classification system developed by Calderón‐Larrañaga et al. [26], in which all International Classification of Disease 10th version (ICD‐10) four‐digit codes were systematically reviewed by a multidisciplinary group and categorized as chronic or non‐chronic. Those deemed chronic were then grouped into 60 clinically meaningful categories based on pathophysiology, prognosis, and treatment considerations.

Multimorbidity was operationalized as the total number of chronic conditions at each wave, based on the 60‐category classification described above. Chronic diseases were further grouped into three organ systems with the greatest impact on disability‐adjusted life years in older adults [27, 28]: (1) cardiovascular diseases, including the following categories: ischemic heart disease, heart failure, atrial fibrillation, cerebrovascular disease, cardiac valve diseases, bradycardias and conduction diseases, peripheral vascular disease, and other cardiovascular diseases; (2) neuropsychiatric diseases, including depression and mood diseases, dementia, neurotic, stress‐related and somatoform diseases, migraine and facial pain syndromes, peripheral neuropathy, Parkinson's disease and parkinsonism, epilepsy, schizophrenia and delusional diseases, multiple sclerosis, and other neuropsychiatric diseases; and (3) musculoskeletal diseases, including dorsopathies, inflammatory arthropathies, osteoarthritis and other degenerative joint diseases, osteoporosis, and other musculoskeletal and joint diseases.

#### Potential confounders

A set of sociodemographic and lifestyle variables was included as potential confounders based on theoretical relations with habitual fasting time and chronic diseases, as well as previous research. Sociodemographic variables included age (continuous), sex (male/female), educational level (elementary, high school, and university), longest held occupation (manual vs. nonmanual), living arrangement (alone vs. not alone), receiving informal care (yes vs. no), and receiving formal care (yes vs. no), all obtained via structured interviews. Social network (composite of social connections and social support tertiles) was derived from the self‐administered questionnaires [29]. Lifestyle and behavioral variables included smoking status (never, former, and current), physical activity during leisure time (inadequate, health‐enhancing, and fitness‐enhancing) [30], and sleep disturbances (none, mild, and moderate to severe) [31], all assessed during nurse interviews. Diet‐related variables included overall diet quality, assessed using the Alternative Healthy Eating Index (tertiles) [32], energy intake (kcal/day, tertiles), total protein intake (g/day, tertiles), and reduced food consumption due to loss of appetite or other factors (self‐reported via the Mini Nutritional Assessment questionnaire; not reduced, moderately, severely reduced) [33]. To retain the full analytical sample, missing indicator categories were created for all covariates except for age and sex, which had no missingness.

### Statistical analyses

#### Descriptive analyses

Descriptive statistics were first generated to summarize baseline characteristics of the study population by categories of habitual fasting duration. Differences in categorical variables were examined using chi‐square tests, and differences in continuous variables were assessed using one‐way analysis of variance.

#### Main analyses

For the main analyses, we used linear mixed models to examine the association between habitual fasting duration and the yearly rate of multimorbidity accumulation over 15 years. These models account for repeated measures within individuals and allow for individual variation in both baseline disease burden and change over time through the inclusion of random intercepts and random slopes, respectively. Time since baseline (in years) was treated as a continuous variable and interacted with habitual fasting duration. This interaction term allowed the model to capture whether the yearly rate of multimorbidity accumulation differed by habitual fasting duration. Positive *β* coefficients indicated faster disease accumulation and vice versa. To assess habitual exposure and reduce measurement error, fasting duration was modeled using a cumulative averaging approach across Waves 1–3, the only waves with available dietary data. For instance, in a participant with three dietary data points, values from Wave 1 were used at the first time point, the average of Waves 1 and 2 was used at the second, and the average of Waves 1, 2, and 3 was used at the third and all subsequent time points. Originally continuous covariates, such as social network and overall diet quality, were modeled using the same approach. For categorical covariates (e.g., smoking status and physical activity), the most recent non‐missing value was used across Waves 1–3 and carried forward in all subsequent observations.

The primary outcome was accumulation of all chronic diseases, with secondary outcomes assessing accumulation within the cardiovascular, neuropsychiatric, and musculoskeletal organ systems. When habitual fasting was modeled continuously, *β* coefficients reflected the change in yearly disease accumulation rates per 1‐h increase in fasting duration. When categorized, these *β* coefficients were interpreted relative to the reference group (6–11.5 h). Three models were used: Model 1 adjusted for age and sex, as well as for the number of chronic diseases unrelated to the disease system being analyzed (e.g., non‐cardiovascular diseases in cardiovascular models); Model 2 further adjusted for other sociodemographic variables, including living arrangement, longest held occupation, education, social network, and reception of informal and formal care; Model 3 further adjusted for health behaviors and dietary characteristics, including smoking status, physical activity, sleep disturbances, dietary quality, energy intake, protein intake, and reduced food consumption.

#### Ancillary analyses

Because age is a key determinant of multimorbidity and dietary habits, we performed age‐stratified associations (<78 vs. ≥78 years), as described in the objectives of the study. To explore potential subgroup differences across levels of other variables, we tested three‐way interaction terms between habitual fasting duration, time from baseline, and each of the 19 potential confounders. A Bonferroni correction for multiple testing was applied, setting the significance threshold for interactions at *p* < 0.0026 [34].

Sensitivity analyses tested the robustness of findings under various conditions: First, we performed a complete case analysis by excluding 779 participants with missing data on any covariate to check whether missing information introduced residual confounding. Second, we analyzed the data using only baseline values for diet and covariates, given that updating these measures over time could not be done for every participant. Third, we alternatively excluded 195 and 343 participants with probable and possible cognitive impairment at baseline, defined as a mini‐mental state examination score ≤24 and <27 (and no updated habitual fasting information when such conditions presented in the follow‐up) [35], to reduce the risk of misclassification bias related to cognitive status. On similar grounds, we excluded participants with dementia at baseline and those who developed this condition on or before the 3‐year follow‐up wave (*n* = 337). Fourth, we excluded 29 participants with implausible energy intake values (>5000 or <800 kcal/day in males and >4000 or <500 kcal/day in females) [36] to minimize bias due to probably misreported dietary information. Fifth, we adjusted for the number of meals per day to account for potential confounding by meal frequency. Sixth, we repeated the analyses using mean instead of maximum habitual fasting duration to assess the sensitivity of the results to fasting operationalization. Seventh, we excluded 34 participants reporting only one and 172 participants reporting only one or two meals per day, given that very low meal frequencies might reflect atypical eating patterns in older populations. Eighth, we excluded 486 participants who reported having meals but did not provide corresponding mealtimes, to account for incomplete habitual fasting duration information. Ninth, we adjusted for breakfast skipping, as breakfast is typically the first major eating occasion of the day, and skipping it may adversely affect health while prolonging the fasting period [37]. Tenth, we analyzed habitual fasting duration using only breakfast, lunch, and dinner to account for the fact that our fasting definition treats all eating occasions equally and does not distinguish between main meals and snacks or drinks, which may not reflect their actual impact on the fasting window. Eleventh, we excluded 140 participants who were not community‐dwelling to account for differences in lifestyle and disease burden among institutionalized older adults. Twelfth, we adjusted for late‐night eating (22:00–4:00), because late food consumption can shorten habitual fasting duration and potentially impact a range of health outcomes [38]. Thirteenth, we adjusted for functional status using the Katz Index of Independence in Activities of Daily Living (ADL) [39] and the Lawton Instrumental Activities of Daily Living Scale (IADL) [40] to address potential reverse causation whereby functional decline may alter eating patterns. Similarly, we adjusted for the frailty phenotype [41], as frail individuals may fast longer due to reduced appetite or functional limitations rather than by choice. Fourteenth, we excluded participants who died before the 3‐year follow‐up wave (*n* = 341) to assess whether early mortality driven by preexisting disease burden may have influenced the observed associations. Fifteenth, we excluded 32 participants with a maximum habitual fasting duration of 8 h or less to assess whether individuals at the lowest extreme of the fasting distribution influenced the results. Sixteenth, we excluded 1643 participants with 4 or more chronic diseases at baseline and stopped updating dietary and covariate information when this threshold was met during follow‐up to examine potential reverse causation from high disease burden. Lastly, we adjusted for the number of total medications (both prescription and nonprescription) to account for potential confounding by polypharmacy [42].

All analyses were performed using *STATA 18 (StataCorp LLC)*.

## Results

### Descriptive results

Characteristics of the study population are shown in Table 1. The mean age at baseline was 74.3 years (standard deviation = 11.0), and 63.8% of participants were female. Participants were followed for a mean of 7.2 years (standard deviation = 5.5); most individuals were followed at least once (73.4%), and more than half were followed twice or more (55.8%). Reasons for non‐participation over time included death, refusal (by participant or proxy), loss of contact, relocation, and occasional cancelled testing (Fig. S1). Missingness was lower than 8% for most covariates (e.g., <2% for education and occupation), but higher for certain lifestyle variables such as energy/protein intake and diet quality (18.4% each). Those with longer habitual fasting durations tended to eat fewer meals, more often skipped breakfast, and reported reduced food consumption; had lower diet quality, energy, and protein intake; and engaged less frequently in physical activity. These participants had a greater burden of chronic diseases (and multimorbidity), used more medications, were older, and were more often female; consistent with this profile, they more often had elementary education, were lifelong manual workers, lived alone, had a lower social network, and more often received informal and formal care.

**Table 1:** Baseline characteristics of the study population, by habitual fasting duration categories (*N* = 2981).

	Habitual fasting categories (hours)				
	6–11.5 h	11.5–12.75 h	12.75–14 h	14–24 h	Total sample
	*N* = 702	*N* = 738	*N* = 884	*N* = 657	*N* = 2981
Meals/Day (*n*)	5.20 (1.00)	4.61 (1.00)	3.98 (0.95)	3.21 (1.00)^a^	4.26 (1.22)
Breakfast					
Skipped	1 (0.1%)	5 (0.7%)	9 (1.0%)	71 (10.8%)	86 (2.9%)
Ate breakfast	701 (99.9%)	733 (99.3%)	875 (99.0%)	586 (89.2%)^a^	2895 (97.1%)
Multimorbidity					
No	100 (14.3%)	111 (15.0%)	94 (10.6%)	47 (7.2%)	352 (11.8%)
Yes	602 (85.8%)	627 (85.0%)	790 (89.4%)	610 (92.9%)^a^	2629 (88.2%)
Chronic conditions (*n*)	3.94 (2.45)	3.90 (2.41)	4.22 (2.41)	4.84 (2.68)^a^	4.21 (2.50)
Total medications (*n*)	3.84 (3.48)	3.57 (3.20)	3.94 (3.21)	4.71 (3.58)^a^	3.99 (3.38)
Age (years)	71.0 (10.3)	72.3 (10.4)	74.9 (10.8)	79.4 (10.8)^a^	74.3 (11.0)
Sex					
Male	269 (38.3%)	266 (36.0%)	339 (38.4%)	205 (31.2%)	1079 (36.2%)
Female	433 (61.7%)	472 (64.0%)	545 (61.7%)	452 (68.8%)^a^	1902 (63.8%)
Living arrangement					
Alone	375 (53.4%)	371 (50.3%)	488 (55.2%)	455 (69.3%)	1689 (56.7%)
Not alone	322 (45.9%)	366 (49.6%)	394 (44.6%)	199 (30.3%)^a^	1281 (43.0%)
Lifelong occupation					
Manual worker	141 (20.1%)	171 (23.2%)	196 (22.2%)	178 (27.1%)	686 (23.0%)
Nonmanual worker	557 (79.3%)	560 (75.9%)	673 (76.1%)	454 (69.1%)^a^	2244 (75.9%)
Education					
Elementary	75 (10.7%)	112 (15.2%)	149 (16.9%)	159 (24.2%)	495 (16.6%)
High school	351 (50.0%)	352 (47.7%)	429 (48.5%)	350 (53.3%)	1482 (49.7%)
University	273 (38.9%)	273 (37.0%)	300 (33.9%)	136 (20.7%)^a^	982 (32.9%)
Social network					
Lower	174 (24.8%)	194 (26.3%)	274 (31.0%	248 (37.8%)	890 (29.9%)
Intermediate	244 (34.8%)	241 (32.7%)	290 (32.8%)	168 (25.6%)	943 (31.6%)
Higher	264 (37.6%)	276 (37.4%)	265 (30.0%)	157 (23.9%)^a^	962 (32.3%)
Informal care					
No	621 (88.5%)	649 (87.9%)	752 (85.1%)	482 (73.4%)	2504 (84.0%)
Yes	76 (10.8%)	82 (11.1%)	115 (13.0%)	144 (21.9%)^a^	417 (14.0%)
Formal care					
No	637 (90.7%)	652 (88.4%)	733 (82.9%)	450 (68.5%)	2472 (82.9%)
Yes	60 (8.6%)	66 (8.9%)	103 (11.7%)	137 (20.9%)^a^	366 (12.3%)
Smoking status					
Never	318 (45.3%)	318 (43.1%)	360 (40.7%)	264 (40.2%)	1260 (42.3%)
Former smoker	278 (39.6%)	292 (39.6%)	314 (35.5%)	200 (30.4%)	1084 (36.4%)
Current smoker	83 (11.8%)	91 (12.3%)	136 (15.4%)	85 (12.9%)	395 (13.3%)
Physical activity					
Inadequate	117 (16.7%)	116 (15.7%)	140 (15.8%)	134 (20.4%)	507 (17.0%)
Health‐enhancing	328 (46.7%)	369 (50.0%)	393 (44.5%)	242 (36.8%)	1332 (44.7%)
Fitness‐enhancing	198 (28.2%)	161 (21.8%)	178 (20.1%)	93 (14.2%)^a^	630 (21.1%)
Sleep disturbances					
None	545 (77.6%)	569 (77.1%)	692 (78.3%)	487 (74.1%)	2293 (76.9%)
Mild	109 (15.5%)	106 (14.4%)	115 (13.0%)	93 (14.2%)	423 (14.2%)
Moderate‐severe	44 (6.3%)	45 (6.1%)	45 (5.1%)	41 (6.2%)	175 (5.9%)
Diet quality					
Lower	196 (27.9%)	178 (24.1%)	232 (26.2%)	192 (29.2%)	798 (26.8%)
Intermediate	207 (29.5%)	221 (30.0%)	254 (28.7%)	135 (20.6%)	817 (27.4%)
Higher	241 (34.3%)	245 (33.2%)	227 (25.6%)	104 (15.8%)^a^	817 (27.4%)
Energy intake (kcal/day)					
Lower	172 (24.5%)	201 (27.2%)	249 (28.2%)	175 (26.6%)	797 (26.7%)
Intermediate	208 (29.6%)	221 (30.0%)	247 (27.9%)	141 (21.5%)	817 (27.4%)
Higher	264 (37.6%)	222 (30.1%)	217 (24.6%)	115 (17.5%)^a^	818 (27.4%)
Protein intake (g/day)					
Lower	182 (25. 9%)	204 (27.6%)	243 (27.5%)	171 (26.0%)	800 (26.8%)
Intermediate	196 (27.9%)	226 (30.6%)	257 (29.1%)	137 (20.9%)	816 (27.4%)
Higher	266 (37.9%)	214 (29.0%)	213 (24.1%)	123 (18.7%)^a^	816 (27.4%)
Reduced food consumption					
Not	647 (92.2%)	687 (93.1%)	784 (88.7%)	555 (84.5%)	2673 (89.7%)
Moderately	48 (6.8%)	41 (5.6%)	80 (9.1%)	78 (11.9%)	247 (8.3%)
Severely	6 (0.9%)	10 (1.4%)	20 (2.3%)	23 (3.5%)^a^	59 (2.0%)

*Note*: Social network (composite of social connections and social support): lower (≤−0.13), intermediate (−0.13 to <0.31), or higher (≥0.31). Physical activity: inadequate (≤2–3 times per month, 2–3 times per month in light and/or moderate/intense exercise), health‐enhancing (light exercise several times per week or every day), or fitness‐enhancing (moderate/intense exercise several times per week or every day). Sleep disturbances: none (0 points), mild (1–2 points), or moderate‐severe (3–6 points), based on the sleep‐related items from the Comprehensive Psychiatric Rating Scale. Diet quality (Alternative Healthy Eating Index): lower (≤57.5 points), intermediate (57.5–65.7 points), or higher (≥65.7 points). Energy intake: lower (≤1653 kcal/day), intermediate (1653–2196 kcal/day), or higher (≥2196 kcal/day). Protein intake: lower (≤55.7 g/day), intermediate (55.7–75.4 g/day), or higher (≥75.4 g/day). The number of participants with missing data was 11 (0.4%) for living arrangement, 51 (1.7%) for occupation, 22 (0.7%) for education, 186 (6.2%) for social network, 60 (2.0%) for informal care, 143 (4.8%) for formal care, 242 (8.1%) for tobacco smoking, 512 (17.2%) for physical activity, 90 (3.0%) for sleep disturbances, 549 (18.4%) for diet quality, 549 (18.4%) for energy intake, 549 (18.4%) for protein intake, and 2 (0.1%) for reduced food consumption. Multimorbidity was defined as having two or more chronic conditions.

^a^Differences across habitual fasting duration categories were statistically significant (*p* < 0.05), based on analysis of variance for continuous variables and chi‐square tests for categorical variables.

### Main results

Table 2 presents the *β* coefficients for the association between habitual fasting duration (by categories and per 1‐h increase) and the annual rate of chronic disease accumulation. Estimates remained largely consistent across models, with slightly weaker associations observed in the fully adjusted model (Model 3). Participants habitually fasting 14–24 h had a 0.119‐U higher annual rate of chronic disease accumulation compared to those fasting 6–11.5 h (*β* = 0.119, 95% confidence interval [CI]: 0.070–0.167). Each 1‐h increase in habitual fasting duration was associated with a 0.019‐U higher annual rate of chronic disease accumulation (95% CI: 0.012–0.027). Figure 1 and Table S1 illustrate the predicted trajectories of total and organ‐system chronic disease accumulation over the 15‐year follow‐up across habitual fasting duration categories. For cardiovascular diseases, the 14–24‐h habitual fasting group had a 0.022‐U higher yearly rate of accumulation (95% CI: 0.006–0.038), although there was not a statistically significant linear relationship (*β* per 1‐h increase = 0.002, 95% CI: −0.001–0.005). For neuropsychiatric diseases, habitual fasting 14–24 h was associated with a 0.019‐U higher accumulation rate (95% CI: 0.005–0.033), and each additional hour of fasting was associated with a significant increase (*β* = 0.005, 95% CI: 0.003–0.007). In contrast, no consistent associations were found for musculoskeletal diseases. A weak, direct association was observed in the 11.5–12.75‐h habitual fasting group (*β* = 0.013, 95% CI: 0.001–0.024), but all other comparisons were nonsignificant, and the linear estimate was zero (*β* per 1‐h increase = 0.000, 95% CI: −0.002–0.003).

**Table 2:** Association between habitual fasting duration and the yearly rate of disease accumulation over a 15‐year follow‐up (*N* = 2981).

			Total chronic diseases (*n* = 60)	Cardiovascular diseases (*n* = 8)	Neuropsychiatric diseases (*n* = 11)	Musculoskeletal diseases (*n* = 5)
		*n*	*β* (95% CI)	*β* (95% CI)	*β* (95% CI)	*β* (95% CI)
Model 1	6–11.5 h	702	Reference	Reference	Reference	Reference
	11.5–12.75 h	738	0.051 (0.011, 0.092)*	0.011 (−0.002, 0.024)	0.004 (−0.008, 0.015)	0.012 (0.001, 0.023)*
	12.75–14 h	884	0.084 (0.043, 0.125)***	0.002 (−0.011, 0.016)	0.017 (0.005, 0.029)**	0.004 (−0.007, 0.015)
	14–24 h	657	0.138 (0.090, 0.186)***	0.027 (0.011, 0.043)***	0.013 (−0.001, 0.027)	0.001 (−0.012, 0.014)
	Per 1 h increase	2981	0.024 (0.016, 0.031)***	0.003 (0.000, 0.005)*	0.005 (0.003, 0.007)***	0.001 (−0.002, 0.003)
Model 2	6–11.5 h	702	Reference	Reference	Reference	Reference
	11.5–12.75 h	738	0.045 (0.006, 0.084)*	0.010 (−0.003, 0.023)	0.003 (−0.009, 0.014)	0.012 (0.001, 0.022)*
	12.75–14 h	884	0.073 (0.033, 0.113)***	0.001 (−0.013, 0.014)	0.015 (0.004, 0.027)**	0.003 (−0.008, 0.014)
	14–24 h	657	0.118 (0.072, 0.164)***	0.023 (0.008, 0.039)**	0.010 (−0.003, 0.024)	−0.000 (−0.013, 0.013)
	Per 1 h increase	2981	0.020 (0.013, 0.028)***	0.002 (−0.000, 0.005)	0.004 (0.002, 0.006)***	0.000 (−0.002, 0.002)
Model 3	6–11.5 h	702	Reference	Reference	Reference	Reference
	11.5–12.75 h	738	0.047 (0.006, 0.088)*	0.012 (−0.002, 0.026)	0.003 (−0.009, 0.015)	0.013 (0.001, 0.024)*
	12.75–14 h	884	0.072 (0.031, 0.114)***	0.001 (−0.013, 0.015)	0.011 (−0.001, 0.023)	0.006 (−0.006, 0.018)
	14–24 h	657	0.119 (0.070, 0.167)***	0.022 (0.006, 0.038)**	0.019 (0.005, 0.033)**	−0.001 (−0.015, 0.012)
	Per 1 h increase	2981	0.019 (0.012, 0.027)***	0.002 (−0.001, 0.005)	0.005 (0.003, 0.007)***	0.000 (−0.002, 0.003)

*Note*: Model 1: Linear mixed model with random intercept and slope, adjusted by age and sex. Models on disease accumulation within a given organ system were adjusted for diseases not belonging to said organ system. Model 2: As Model 1, additionally adjusted by living arrangements, lifelong occupation, education, social network, informal care, and formal care. Model 3: As Model 2, additionally adjusted by smoking status, physical activity, energy intake, sleep disturbances, diet quality, protein intake, and reduced food consumption.

Abbreviation: CI, confidence interval.

*p value < 0.05.

**p value < 0.01.

***p value < 0.001.

**Fig. 1:**
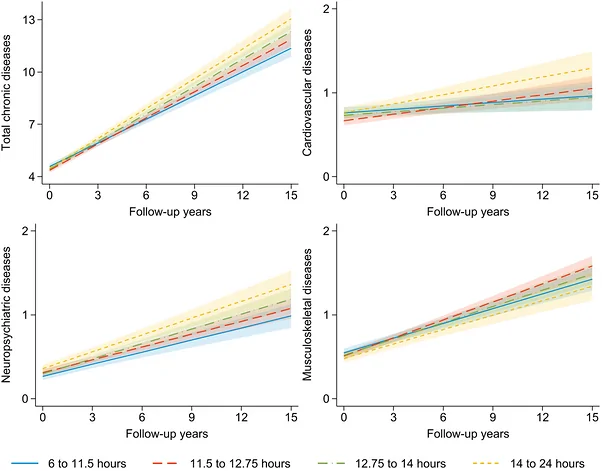
Predicted trajectories of total chronic disease accumulation over a 15‐year follow‐up across habitual fasting duration categories (*N* = 2981). Linear mixed model with random intercept and slope, adjusted as Model 3 in Table 2: age, sex, living arrangements, previous occupation, education, social network, informal care, formal care, smoking status, physical activity, energy intake, sleep disturbance, diet quality, protein intake, and reduced food consumption. Models on disease accumulation within a given organ system were adjusted for diseases not belonging to said organ system. Shaded areas represent 95% confidence intervals.

We also explored whether the association between habitual fasting duration and disease accumulation varied by age (Table 3). In the older age group (≥78 years), longer habitual fasting durations were significantly associated with faster accumulation of total chronic diseases. Specifically, participants habitually fasting 14–24 h had a 0.099‐U higher yearly rate of multimorbidity accumulation (95% CI: 0.031–0.168) compared to those fasting 6–11.5 h, and each additional hour of habitual fasting was associated with a 0.014‐U increase in annual chronic disease accumulation (95% CI: 0.003–0.026). Among younger participants (<78 years), no statistically significant associations were observed for the highest habitual fasting category (*β* = −0.009, 95% CI: −0.078, 0.061, *p* for age interaction: 0.030) or linearly (*β* per 1‐h increase = 0.004, 95% CI: −0.007 to 0.014, *p* for interaction: 0.175) in relation to total multimorbidity. Figure 2 illustrates the predicted trajectories of total chronic disease accumulation over the 15‐year follow‐up across habitual fasting duration categories, stratified by age. Similar age‐specific patterns were seen for neuropsychiatric diseases, where the association per 1‐h increase in habitual fasting time was significant only in the older age group (*β* = 0.006, 95% CI: 0.002–0.009 vs. 0.001 [−0.002, 0.004], *p* for age interaction: 0.064). No associations between habitual fasting duration and cardiovascular or musculoskeletal diseases were apparent in the age‐stratified analyses.

**Table 3:** Association between habitual fasting duration and the yearly rate of disease accumulation over a 15‐year follow‐up, stratified by age (<78 years = 1619, ≥78 years = 1362).

			Total chronic diseases (*n* = 60)	Cardiovascular diseases (*n* = 8)	Neuropsychiatric diseases (*n* = 11)	Musculoskeletal diseases (*n* = 5)
		*n*	*β* (95% CI)	*β* (95% CI)	*β* (95% CI)	*β* (95% CI)
<78 years	6–11.5 h	471	Reference	Reference	Reference	Reference
	11.5–12.75 h	455	0.022 (−0.027, 0.070)	0.012 (−0.004, 0.029)	−0.002 (−0.016, 0.013)	0.010 (−0.005, 0.024)
	12.75–14 h	469	0.011 (−0.040, 0.061)	0.001 (−0.016, 0.018)	0.004 (−0.011, 0.019)	−0.000 (−0.015, 0.015)
	14–24 h	224	−0.009 (−0.078, 0.061)	0.010 (−0.014, 0.034)	−0.009 (−0.030, 0.011)	−0.001 (−0.021, 0.020)
	Per 1 h increase	1619	0.004 (−0.007, 0.014)	0.001 (−0.003, 0.005)	0.001 (−0.002, 0.004)	0.000 (−0.003, 0.003)
≥78 years	6–11.5 h	231	Reference	Reference	Reference	Reference
	11.5–12.75 h	283	0.055 (−0.011, 0.121)	0.005 (−0.019, 0.028)	0.003 (−0.017, 0.023)	0.017 (−0.003, 0.037)
	12.75–14 h	415	0.091 (0.026, 0.156)**	−0.014 (−0.036, 0.009)	0.010 (−0.010, 0.029)	0.010 (−0.009, 0.030)
	14–24 h	433	0.099 (0.031, 0.168)**	−0.002 (−0.026, 0.022)	0.021 (0.000, 0.042)*	−0.004 (−0.024, 0.017)
	Per 1 h increase	1362	0.014 (0.003, 0.026)*	−0.002 (−0.006, 0.002)	0.006 (0.002, 0.009)***	−0.000 (−0.004, 0.003)

*Note*: Linear mixed model with random intercept and slope, adjusted as Model 3 in Table 2: age, sex, living arrangements, previous occupation, education, social network, informal care, formal care, smoking status, physical activity, energy intake, sleep disturbance, diet quality, protein intake, and reduced food consumption. Models on disease accumulation within a given organ system were adjusted for diseases not belonging to said organ system. Estimates were obtained from models with multiplicative interaction terms among habitual fasting duration, time from baseline, and age (<78, ≥78 years).

Abbreviation: CI, confidence interval.

*p value < 0.05.

**p value < 0.01.

***p value < 0.001.

**Fig. 2:**
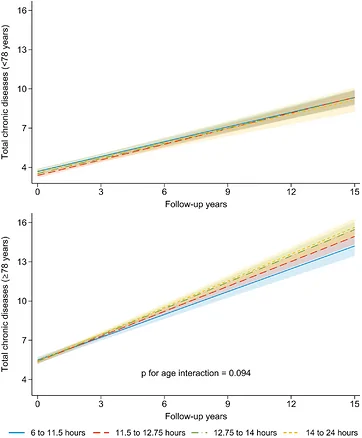
Predicted trajectories of total chronic disease accumulation over a 15‐year follow‐up across habitual fasting duration categories, stratified by age (<78 years = 1619, ≥78 years = 1362). Linear mixed model with random intercept and slope, adjusted as in Table 3: age, sex, living arrangements, previous occupation, education, social network, informal care, formal care, smoking status, physical activity, energy intake, sleep disturbance, diet quality, protein intake, and reduced food consumption. Estimates were obtained from models with multiplicative interaction terms among habitual fasting duration, time from baseline, and age (<78, ≥78 years). The p‐value shown represents the global test of this interaction. Shaded areas represent 95% confidence intervals.

### Ancillary analyses

We also tested all three‐way interaction terms between habitual fasting duration, time from baseline, and each covariate included in the models. For total multimorbidity and cardiovascular diseases, no statistically significant interactions were observed. For neuropsychiatric diseases, three interactions reached nominal significance: tobacco use (*p* = 0.045), formal care reception (*p* = 0.025), and IADL (*p* = 0.004). Stratified estimates indicated that the association between prolonged habitual fasting and neuropsychiatric multimorbidity was only observed among never smokers, participants receiving formal care, and those with severe IADL dependence. For musculoskeletal diseases, nominal significance was observed for lifelong occupation (*p* = 0.028) and diet quality (*p* = 0.013) interactions, with associations only among participants with manual occupations and those with higher diet quality. However, none of the interactions remained significant after correction for multiple testing. Results are presented in Fig. S2.

As shown in Table S2, sensitivity analyses produced consistent findings under 21 different modeling assumptions. The linear associations of longer habitual fasting time with faster total and neuropsychiatric chronic disease accumulation remained stable in most sensitivity analyses, and there were even three instances where an association with cardiovascular diseases became apparent.

## Discussion

In this cohort of older Swedish adults, longer habitual fasting durations were associated with significantly faster accumulation of total, cardiovascular, and neuropsychiatric chronic diseases, particularly among the older old. These associations were not meaningfully altered by adjustment for sociodemographic, lifestyle, or dietary factors and remained robust across multiple sensitivity analyses.

### Interpretation

Previous studies in younger populations reported metabolic benefits of fasting, such as improved insulin sensitivity and reduced inflammation [43, 44], and a recent network meta‐analysis of 99 randomized controlled trials showed modest weight loss and improvements in cardiometabolic risk factors with intermittent fasting strategies when compared to unrestricted eating [45]. Conversely, our findings suggest that extended habitual fasting windows may be detrimental for older adults. Associations were strongest in participants aged 78 and older, whereas little evidence of an association was observed in younger old groups, indicating an age‐related vulnerability. Similar to our findings, a recent longitudinal study found that a later breakfast and shorter eating windows were associated with greater multimorbidity among older adults [46]. However, a cross‐sectional study in Italy found that early dinner timing combined with a prolonged overnight fasting period of approximately 17.5 h was a common dietary pattern among individuals who survived to extreme old age [47]. Several factors may explain why these findings differ from those observed in the present study. First, studies of centenarians describe dietary patterns of a selected survivor cohort, whereas the present study prospectively assessed the rate of chronic disease accumulation in a general older population (note how we did not observe any associations among potentially selected subgroups, e.g., smokers). Second, multimorbidity accumulation is a continuous, non‐saturable outcome and conceptually distinct from survival to extreme old age—which likely reflects a combination of genetic, environmental, and behavioral factors that may not generalize to the broader aging population.

In the general older population studied here, one plausible explanation lies in age‐related declines in digestive capacity and nutrient absorption. Even with adequate energy and protein intake and diet quality, reduced gastric acid secretion and pancreatic enzyme activity can impair absorption of key micronutrients such as vitamin B12, folate, calcium, and iron [22, 23]. Additional changes in gastrointestinal function, including delayed gastric emptying, altered motility, and microbiota shifts, may further limit nutrient absorption [48, 49]. In the context of prolonged habitual fasting, which compresses the eating window, this reduced absorption capacity could contribute to subtle but cumulative nutrient deficiencies [21], which may in turn contribute to chronic disease accumulation. In older adults, lower dietary micronutrient adequacy is associated with a higher risk of developing multimorbidity [50]. Thus, our observed associations between longer habitual fasting duration and faster multimorbidity accumulation (particularly in the older old) may reflect an underlying interaction between reduced nutrient absorption capacity and the physiological demands of prolonged habitual fasting in already vulnerable individuals. Although no studies, to our knowledge, have directly tested this interaction, related findings suggest that prolonged fasting may be less beneficial, or even detrimental, for older individuals. Two cross‐sectional studies of community‐dwelling older adults in Spain found that prolonged habitual overnight fasting (greater than 12 h) in older adults was associated not only with adverse metabolic biomarkers but also with significantly increased odds of impaired lower extremity function [18, 19]. This may reflect age‐related anabolic resistance, whereby older muscle responds less efficiently to dietary protein. Longer fasting intervals reduce the number of anabolic stimulation opportunities and limit an even protein distribution across meals, both of which have been linked to lower skeletal muscle mass in older adults [51]. Sarcopenia (the age‐related loss of skeletal muscle mass and function) has, in turn, been linked to adverse health outcomes spanning multiple organ systems, including cardiovascular disease, metabolic disorders, and cognitive impairment [52], offering a plausible pathway through which prolonged habitual fasting could contribute to the accumulation of chronic diseases, particularly in the older old. Zhang et al. [16] reported a U‐shaped association between habitual fasting duration and mortality in older adults, with both short and prolonged fasting periods linked to increased cardiovascular mortality. Age‐related declines in insulin sensitivity, *β*‐cell responsiveness, and skeletal muscle glucose uptake may also reduce metabolic flexibility, potentially making older individuals more susceptible to blood glucose fluctuations during prolonged fasting [53]. Moreover, many commonly prescribed drugs require regular food consumption to ensure proper absorption or minimize adverse effects (e.g., NSAIDs, sulfonylureas) [54], and older adults are disproportionately exposed to polypharmacy [55]. Prolonged habitual fasting may therefore interfere with drug‐related food requirements, potentially increasing the risk of adverse effects—as suggested by our interaction analysis. A prior systematic review concluded that robust human evidence for the metabolic benefits of fasting remains limited, and that prolonged or frequent fasting may have adverse effects, including malnutrition and susceptibility to infection, warranting caution in older adults [20].

In our study, we observed consistent associations between longer habitual fasting duration and faster accumulation of total, cardiovascular, and neuropsychiatric diseases. However, no clear associations were observed with musculoskeletal multimorbidity in any model. This absence of association aligns with findings from other studies in the SNAC‐K cohort, where dietary patterns were not associated with musculoskeletal multimorbidity [9], and the latter was in turn not associated with adverse outcomes such as cognitive decline [56], physical frailty [57], institutionalization [58], or dementia incidence [59]. One possible explanation is that musculoskeletal diseases may contribute less to clinical deterioration in later life in this population, or that their effects become smaller when grouped into broader multimorbidity organ systems. Alternatively, musculoskeletal conditions may constitute a more stable or slowly progressing organ system, less responsive to short‐ or medium‐term behavioral exposures such as habitual fasting. Finally, anti‐inflammatory effects of fasting may be more consequential for musculoskeletal conditions [60, 61], potentially offsetting other drawbacks and mitigating deleterious associations in this domain.

Although none of the tested three‐way interactions remained statistically significant after correction for multiple testing, some subgroup analyses suggested that associations between habitual fasting duration and multimorbidity accumulation might differ across levels of third variables. For neuropsychiatric multimorbidity, longer habitual fasting durations appeared more detrimental among never smokers, individuals receiving formal care, and those with severe IADL dependence. The stronger association among never smokers may reflect selection bias, whereby ever smokers surviving into old age represent a more resilient subgroup [62], whereas the associations among those receiving formal care and with severe functional limitations may instead reflect greater underlying vulnerability [58, 63]. For musculoskeletal multimorbidity, associations were more pronounced among participants with manual occupations and those with higher diet quality, which may reflect reduced physical resilience [64] and lower competing nutritional risk, respectively.

### Generalizability

In older adults, meal timing and frequency are often shaped by social circumstances such as living arrangements, caregiving context, and daily routines, which may, in turn, independently influence chronic disease trajectories [65]. To examine whether social factors modified the observed associations, several social determinants, including living arrangement, lifelong occupation and education (as proxies for socioeconomic position), formal and informal care reception, and social network, were tested as potential effect modifiers. None of the corresponding interaction terms reached statistical significance after multiple comparisons correction, suggesting that the association between habitual fasting duration and multimorbidity accumulation did not meaningfully differ across levels of social position in this cohort.

The generalizability of our study may be further limited by the characteristics of the SNAC‐K cohort, which consisted predominantly of highly educated, urban‐dwelling, and community‐living older adults in Sweden. Moreover, fasting behaviors and their health consequences may differ in younger adults, who may better tolerate longer habitual fasting [12, 13, 14], and in disadvantaged or rural populations, where irregular eating patterns may reflect limited food access or labor demands rather than voluntary behaviors. Institutionalized older adults, who were sparsely represented in our sample and often follow structured meal schedules [66, 67], may also show different associations. In addition, cultural fasting practices and healthcare infrastructures vary across countries, influencing both exposures and outcomes. Caution is therefore warranted when extrapolating these findings beyond similar high‐income, community‐dwelling European populations.

### Limitations

Several limitations should be considered. Information on habitual fasting duration and many potential confounders were self‐reported, which may have introduced some misclassification. In addition, we lacked mealtime data prior to baseline, which restricts our capacity to assess earlier life dietary habits. Moreover, our fasting definition (maximum gap between any two eating occasions) assumes equal metabolic impact for all meals. A late‐night snack was considered equivalent to a full dinner, which may not reflect their actual impact on the fasting window. Additionally, our habitual fasting categories were based on approximate quartiles to ensure adequate group sizes, rather than on predefined metabolic thresholds. As a result, the reference group (6–11.5 h) includes intervals that likely reflect normal meal spacing rather than a physiologically meaningful fasting state, whereas most participants (76.5%) had habitual fasting durations exceeding 11.5 h, consistent with culturally typical overnight fasting in Sweden. By also modeling habitual fasting duration continuously and conducting a sensitivity analysis excluding participants with fasting durations of 8 h or less, we were able to assess associations across the full range of habitual fasting time while addressing concerns about shorter intervals. This data‐driven approach may nonetheless limit comparability with studies using predefined fasting thresholds. Moreover, we do not know if participants engaged in fasting intentionally. Thus, we could not distinguish between voluntary fasting patterns and involuntary meal skipping due to factors such as illness, appetite loss, or socioeconomic circumstances. It is of note that the main models already adjusted for several variables that may proxy for non‐intentional habitual fasting, including social network, formal and informal care reception, and reduced food consumption. To address this concern further, we conducted sensitivity analyses adjusting for functional status (ADL and IADL dependence) and frailty, which may proxy for involuntary habitual fasting due to physical limitation or vulnerability. Associations remained consistent in these models, suggesting that results were not primarily driven by non‐intentional habitual fasting related to functional decline.

As with any observational study, the possibility of reverse causation cannot be ruled out. In other words, individuals may have adjusted their eating patterns as a result of their greater disease burden. In the main analyses, missing data on follow‐up habitual fasting duration and covariates were addressed by carrying forward the nearest available value from earlier waves. Although this approach helped reduce missingness, it assumed stability of behaviors and exposures over multi‐year intervals, which may not reflect true changes and could introduce some misclassification. Nevertheless, several of these limitations were addressed in sensitivity analyses, which yielded consistent results.

Although linear mixed models account for baseline differences in multimorbidity when estimating trajectories, potential selection bias due to differential survival or follow‐up participation across habitual fasting categories cannot be entirely ruled out. Because we examined a large number of potential interactions, and none remained statistically significant after correction for multiple testing, subgroup analyses other than age‐stratified associations should be viewed as exploratory and interpreted with caution.

## Conclusion

In this 15‐year longitudinal study of Swedish older adults, longer habitual fasting durations were associated with faster accumulation of total, cardiovascular, and neuropsychiatric chronic diseases, particularly among participants aged 78 and older. No clear associations were observed for musculoskeletal multimorbidity in either age group. Although fasting is often promoted for metabolic health, our findings suggest that such benefits may be confined to younger individuals.

## Author contributions

**Luis Kalmbach**: Writing—original draft; visualization; writing—review and editing; conceptualization; data curation; formal analysis. **David Abbad‐Gomez**: Writing—review and editing; methodology; formal analysis; visualization. **Giorgi Beridze**: Writing—review and editing; visualization; data curation. **Fernando Rodríguez‐Artalejo**: Conceptualization; writing—review and editing. **Davide Liborio Vetrano**: Conceptualization; writing—review and editing; data curation. **Amaia Calderón‐Larrañaga**: Funding acquisition; writing—review and editing, data curation. **Adrián Carballo‐Casla**: Funding acquisition; conceptualization; writing—original draft; writing—review and editing; visualization; methodology; formal analysis; project administration; resources; supervision; data curation.

## Conflict of interest statement

The authors declare no conflicts of interest.

## Funding information

Data collection of the Swedish National study on Aging and Care (SNAC‐K) was supported by the Swedish Research Council (ongoing/current grant: 2021‐00178); the Swedish Ministry of Health and Social Affairs; the participating County Councils and Municipalities. Davide Liborio Vetrano was supported by the Swedish Research Council (project number 2021‐03324) and the Karolinska Institutet Strategic Research Area in Epidemiology and Biostatistics (SFOepi) in 2021 and 2023 and the Karolinska Institutet Strategic Research Area in Neuroscience in 2025. Amaia Calderón‐Larrañaga received funding from the Swedish Research Council (project number 2021‐06398), the Swedish Research Council for Health, Working Life and Welfare (project numbers 2024‐01830 and 2021‐00256), Karolinska Institutet's Strategic Research Area in Epidemiology and Biostatistics SFOepi (consolidator bridging grant, 2023), and Alzheimerfonden (AF‐1010573, 2024). Adrián Carballo‐Casla received funding from the Foundation for Geriatric Diseases at Karolinska Institutet (project number 2024:0011); the Karolinska Institutet Research Foundation Grants (project number 2024:0017); the David and Astrid Hagelén Foundation (project number 2024:0005); and the Swedish Research Council for Health, Working Life and Welfare (project number STY‐2024/0005).

## Supporting information




**Table S1**. Total and system‐specific number of chronic diseases at the 3, 6, 9, 12, and 15‐year follow‐ups, by habitual fasting duration categories (N = 2,981).
**Table S2**. Sensitivity analyses of the associations between habitual fasting duration and the yearly rate of chronic disease accumulation over a 15‐year follow‐up.
**Figure S1**. Flow chart of the study participants.
**Figure S2**. Association between habitual fasting duration and the yearly rate of chronic disease accumulation during a 15‐year follow‐up, stratified by selected covariates.

## Data Availability

Data are from the SNAC‐K project, a population‐based study designed to investigate aging processes and identify preventive strategies to enhance health and care in older adults (https://www.snac‐k.se/). Access to the original data is available to the research community upon approval by the SNAC‐K organization. Applications for accessing these data can be submitted through https://www.snac‐k.se/for‐researchers/application‐form/.
