# Supplementary material for: Habitual fasting duration and accelerated multimorbidity in older adults
Source: J Intern Med. 2026 Aug 20;300(5):642–58. doi: 10.1111/joim.70150 (PMC13641277; doi:10.1111/joim.70150)
Supplement: Supplementary file 1 [file JOIM-300-642-s001.docx]

Supporting information

Title: **Habitual fasting duration and accelerated multimorbidity in older adults**

Short title: **Fasting duration and multimorbidity**

**Author list**

Luis Kalmbach, MPH,^1^ David Abbad-Gomez, MD,^2,3,4,5^ Giorgi Beridze, MD, PhD,^1^ Fernando Rodríguez-Artalejo, MD, PhD,^6,7,8^ Davide Liborio Vetrano, MD, PhD,^1,9^ Amaia Calderón-Larrañaga, PhD,^1,9^ Adrián Carballo-Casla, PhD^1,7^

**Affiliations**

1: Aging Research Center, Department of Neurobiology, Care Sciences and Society, Karolinska Institutet and Stockholm University, Stockholm, Sweden.

2: Department of Epidemiology and Evaluation, Hospital del Mar, Barcelona, Spain

3: Hospital del Mar Research Institute, Barcelona, Spain

4: Research Network on Chronicity, Primary Care and Health Promotion (RICAPPS), Instituto de Salud Carlos III (ISCIII) Madrid, Spain

5: PhD program in Biomedicine, Universitat Pompeu Fabra, Barcelona, Spain.

6: Department of Preventive Medicine and Public Health, Universidad Autónoma de Madrid, Madrid, Spain.

7: Center for Networked Biomedical Research in Epidemiology and Public Health (CIBERESP), Madrid, Spain.

8: IMDEA Institute of Nutrition, Campus of International Excellence, Madrid, Spain.

9: Stockholm Gerontology Research Center, Stockholm, Sweden.

**Corresponding author**

Adrián Carballo-Casla: Widerströmska huset, floor 10. Tomtebodavägen 18a, 171 65 Solna. adrian.carballo.casla@ki.se

**Table S1.** Total and system-specific number of chronic diseases at the 3, 6, 9, 12, and 15-year follow-ups, by habitual fasting duration categories (N = 2,981)

|  |  | | 3-year follow-up | 6-year follow-up | 9-year follow-up | 12-year follow-up | 15-year follow-up |
| --- | --- | --- | --- | --- | --- | --- | --- |
|  |  | n | Estimate (95% CI) | Estimate (95% CI) | Estimate (95% CI) | Estimate (95% CI) | Estimate (95% CI) |
| Total chronic diseases (n=60) | 6 to 11.5 h | 702 | 5.93 (5.76, 6.11) | 7.29 (7.06, 7.53) | 8.65 (8.34, 8.97) | 10.01 (9.61, 10.42) | 11.37 (10.88, 11.87) |
|  | 11.5 to 12.75 h | 738 | 5.87 (5.73, 6.01) | 7.37 (7.18, 7.56) | 8.87 (8.62, 9.13) | 10.37 (10.04, 10.71) | 11.88 (11.46, 12.29) |
|  | 12.75 to 14 h | 884 | 6.02 (5.87, 6.16) | 7.59 (7.40, 7.79) | 9.17 (8.90, 9.44) | 10.75 (10.40, 11.09) | 12.32 (11.90, 12.75) |
|  | 14 to 24 h | 657 | 6.19 (6.01, 6.36) | 7.90 (7.65, 8.16) | 9.62 (9.27, 9.98) | 11.34 (10.87, 11.80) | 13.05 (12.48, 13.63) |
| Cardiovascular diseases (n=8) | 6 to 11.5 h | 702 | 0.80 (0.74, 0.87) | 0.84 (0.76, 0.92) | 0.88 (0.77, 0.99) | 0.92 (0.78, 1.06) | 0.96 (0.72, 1.13) |
|  | 11.5 to 12.75 h | 738 | 0.75 (0.69, 0.80) | 0.82 (0.76, 0.89) | 0.90 (0.81, 0.99) | 0.98 (0.86, 1.09) | 1.05 (0.91, 1.20) |
|  | 12.75 to 14 h | 884 | 0.77 (0.72, 0.83) | 0.82 (0.75, 0.89) | 0.86 (0.77, 0.95) | 0.90 (0.78, 1.02) | 0.94 (0.79, 1.10) |
|  | 14 to 24 h | 657 | 0.87 (0.80, 0.93) | 0.98 (0.89, 1.06) | 1.08 (0.96, 1.21) | 1.19 (1.03, 1.35) | 1.29 (1.09, 1.50) |
| Neuropsychiatric diseases (n=11) | 6 to 11.5 h | 702 | 0.41 (0.36, 0.46) | 0.56 (0.49, 0.62) | 0.70 (0.61, 0.79) | 0.84 (0.73, 0.96) | 0.99 (0.85, 1.13) |
|  | 11.5 to 12.75 h | 738 | 0.46 (0.42, 0.50) | 0.62 (0.56, 0.67) | 0.77 (0.70, 0.84) | 0.92 (0.83, 1.02) | 1.08 (0.96, 1.19) |
|  | 12.75 to 14 h | 884 | 0.48 (0.44, 0.51) | 0.65 (0.60, 0.71) | 0.83 (0.75, 0.91) | 1.01 (0.91, 1.11) | 1.18 (1.06, 1.31) |
|  | 14 to 24 h | 657 | 0.56 (0.51, 0.61) | 0.76 (0.69, 0.83) | 0.96 (0.86, 1.06) | 1.16 (1.03, 1.30) | 1.36 (1.19, 1.53) |
| Musculoskeletal diseases (n=5) | 6 to 11.5 h | 702 | 0.72 (0.67, 0.77) | 0.90 (0.83, 0.96) | 1.07 (0.99, 1.16) | 1.25 (1.14, 1.36) | 1.43 (1.29, 1.56) |
|  | 11.5 to 12.75 h | 738 | 0.72 (0.68, 0.77) | 0.94 (0.89, 0.99) | 1.15 (1.08, 1.23) | 1.37 (1.27, 1.46) | 1.58 (1.47, 1.70) |
|  | 12.75 to 14 h | 884 | 0.71 (0.67, 0.75) | 0.91 (0.85, 0.96) | 1.10 (1.02, 1.18) | 1.29 (1.19, 1.39) | 1.49 (1.36, 1.61) |
|  | 14 to 24 h | 657 | 0.65 (0.60, 0.70) | 0.82 (0.75, 0.90) | 1.00 (0.89, 1.10) | 1.17 (1.03, 1.30) | 1.34 (1.17, 1.51) |

Linear mixed model with random intercept and slope, adjusted as Model 3 in Table 2: age, sex, living arrangements, previous occupation, education, social network, informal care, formal care, smoking status, physical activity, energy intake, sleep disturbance, diet quality, protein intake, and reduced food consumption. Models on disease accumulation within a given organ system were adjusted for diseases not belonging to said organ system.

**Table S2**. Sensitivity analyses of the associations between habitual fasting duration and the yearly rate of chronic disease accumulation over a 15-year follow-up

|  | Total chronic disease | | Cardiovascular diseases | | Neuropsychiatric diseases | | Musculoskeletal diseases | |
| --- | --- | --- | --- | --- | --- | --- | --- | --- |
|  | β (95%CI) | | β (95%CI) | | β (95%CI) | | β (95%CI) | |
| Complete case analysis (n = 2202) | 0.016 (0.007, 0.025) | *** | 0.003 (-0.000, 0.006) |  | 0.002 (-0.000, 0.005) |  | 0.001 (-0.002, 0.003) |  |
| Using only baseline data on diet and covariables (n = 2981) | 0.018 (0.011, 0.024) | *** | 0.002 (-0.001, 0.004) |  | 0.003 (0.001, 0.005) | ** | 0.001 (-0.001, 0.003) |  |
| Excluding participants with MMSE ≤24 (n = 2786) | 0.018 (0.010, 0.026) | *** | 0.002 (-0.001, 0.005) |  | 0.004 (0.002, 0.007) | *** | 0.000 (-0.002, 0.003) |  |
| Excluding participants with MMSE ≤27 (n = 2638) | 0.019 (0.010, 0.027) | *** | 0.002 (-0.001, 0.004) |  | 0.003 (0.001, 0.006) | ** | 0.001 (-0.002, 0.003) |  |
| Excluding participants with dementia in the first 3 years (n= 2644) | 0.017 (0.009, 0.026) | *** | 0.002 (-0.000, 0.005) |  | 0.003 (0.001, 0.005) | * | 0.001 (-0.002, 0.003) |  |
| Excluding probable diet misreporters (n = 2952) | 0.022 (0.014, 0.029) | *** | 0.003 (0.000, 0.005) | * | 0.003 (0.001, 0.005) | ** | 0.002 (-0.000, 0.003) |  |
| Adjusting for number of meals  (n = 2981) | 0.019 (0.011, 0.027) | *** | 0.002 (-0.001, 0.005) |  | 0.005 (0.003, 0.007) | *** | 0.000 (-0.002, 0.003) |  |
| Analyzing mean fasting time instead of maximum fasting time  (n = 2981) | 0.012 (0.004, 0.019) | ** | 0.002 (0.000, 0.005) | * | 0.005 (0.003, 0.007) | *** | -0.000 (-0.002, 0.002) |  |
| Excluding participants with one meal (n = 2947) | 0.019 (0.010, 0.028) | *** | 0.002 (-0.001, 0.005) |  | 0.005 (0.002, 0.007) | *** | -0.000 (-0.003, 0.002) |  |
| Excluding participants with one or two meals (n = 2809) | 0.024 (0.014, 0.034) | *** | 0.002 (-0.001, 0.006) |  | 0.005 (0.002, 0.008) | *** | 0.000 (-0.003, 0.003) |  |
| Excluding participants reporting meals but not mealtimes (n = 2495) | 0.023 (0.014, 0.032) | *** | 0.002 (-0.001, 0.005) |  | 0.006 (0.003, 0.009) | *** | 0.001 (-0.002, 0.003) |  |
| Adjusted for breakfast skipping (n = 2981) | 0.019 (0.011, 0.027) | *** | 0.002 (-0.001, 0.005) |  | 0.005 (0.003, 0.007) | *** | 0.000 (-0.002, 0.003) |  |
| Analyzing maximum fasting time considering only breakfast, lunch, and dinner (n = 2981) | 0.023 (0.015, 0.031) | *** | 0.005 (0.002, 0.008) | *** | 0.006 (0.003, 0.008) | *** | -0.001 (-0.004, 0.001) |  |
| Excluding institutionalized participants (n = 2841) | 0.019 (0.011, 0.028) | *** | 0.002 (-0.000, 0.005) |  | 0.004 (0.002, 0.007) | *** | 0.000 (-0.002, 0.002) |  |
| Adjusting for night eating (n = 2981) | 0.019 (0.012, 0.027) | *** | 0.002 (-0.001, 0.005) |  | 0.005 (0.003, 0.007) | *** | 0.000 (-0.002, 0.003) |  |
| Adjusting for ADL and IADL (n = 2901) | 0.017 (0.009, 0.025) | *** | 0.001 (-0.001, 0.004) |  | 0.004 (0.002, 0.006) | *** | 0.000 (-0.002, 0.002) |  |
| Adjusting for frailty (n= 2981) | 0.018 (0.010, 0.026) | *** | 0.002 (-0.001, 0.004) |  | 0.005 (0.003, 0.007) | *** | 0.000 (-0.002, 0.003) |  |
| Excluding participants who died in the first 3 years (n= 2640) | 0.019 (0.012, 0.027) | *** | 0.002 (-0.000, 0.005) |  | 0.005 (0.003, 0.007) | *** | 0.000 (-0.002, 0.003) |  |
| Excluding participants who fasted ≤8 hours (n= 2949) | 0.019 (0.011, 0.027) | *** | 0.002 (-0.001, 0.005) |  | 0.005 (0.003, 0.007) | *** | 0.000 (-0.002, 0.003) |  |
| Excluding participants with ≥4 chronic diseases (n= 1338) | 0.016 (0.006, 0.026) | ** | 0.002 (-0.001, 0.005) |  | 0.002 (-0.000, 0.005) |  | 0.001 (-0.002, 0.004) |  |
| Adjusting for number of total medications (n= 2978) | 0.014 (0.007, 0.022) | *** | 0.002 (-0.001, 0.004) |  | 0.005 (0.003, 0.007) | *** | 0.000 (-0.002, 0.002) |  |

MMSE: Mini-Mental State Examination. ADL: Katz Index of Independence in Activities of Daily Living. IADL: Lawton Instrumental Activities of Daily Living. CI: confidence interval. Habitual fasting duration was operationalized as maximum fasting time unless otherwise specified.

Linear mixed model with random intercept and slope, adjusted as Model 3 in Table 2: age, sex, living arrangements, previous occupation, education, social network, informal care, formal care, smoking status, physical activity, energy intake, sleep disturbance, diet quality, protein intake, and reduced food consumption. Models on disease accumulation within a given organ system were adjusted for diseases not belonging to said organ system.

**Figure S1.** Flow chart of the study participants.

**Figure S2**. Association between habitual fasting duration and the yearly rate of chronic disease accumulation during a 15-year follow-up, stratified by selected covariates.

Linear mixed model with random intercept and slope, adjusted as Model 3 in Table 2: age, sex, living arrangements, previous occupation, education, social network, informal care, formal care, smoking status, physical activity, energy intake, sleep disturbance, diet quality, protein intake, and reduced food consumption. Models on disease accumulation within a given organ system were adjusted for diseases not belonging to said organ system.

Estimates were obtained from models with multiplicative interaction terms among maximum fasting duration, time from baseline, and the stratification variables.

P-values for the interaction terms are shown. Given the multiple interaction tests, a Bonferroni correction would set the significance threshold at p < 0.0026.
